# Utility of urinary liver-type fatty acid-binding protein as a predictor of renal dysfunction in Japanese patients with HIV receiving tenofovir disoproxil fumarate with low urinary β2 microglobulin levels: a retrospective observational study

**DOI:** 10.1186/s40780-019-0140-8

**Published:** 2019-06-06

**Authors:** Shinichi Hikasa, Shota Shimabukuro, Kyoko Hideta, Satoshi Higasa, Akihiro Sawada, Tazuko Tokugawa, Kuniyoshi Tanaka, Mina Yanai, Takeshi Kimura

**Affiliations:** 1 Department of pharmacy, The Hospital of Hyogo College of Medicine, 1-1, Mukogawa-cho, Nishinomiya, Hyogo 663-8501 Japan; 2 Division of Hematology, Department of Internal Medicine, Hyogo College of Medicine, 1-1, Mukogawa-cho, Nishinomiya, Hyogo 663-8501 Japan

**Keywords:** Tenofovir, L-FABP, HIV, Renal dysfunction

## Abstract

**Background:**

Tenofovir disoproxil fumarate (TDF) is known to reduce estimated glomerular filtration rate (eGFR). It is clinically important to identify patients at high risk for renal dysfunction as early as possible. Among the tubular markers, urinary β2 microglobulin (Uβ2MG) is a well-known biomarker of TDF-related tubulopathy. However, renal dysfunction has often been occurred in patients receiving TDF with low Uβ2MG levels. Recently, urinary liver-type fatty acid–binding protein (UL-FABP) was suggested to be predictor of the progression of renal dysfunction. Thus, we focused on UL-FABP in patients receiving TDF with low Uβ2MG levels.

**Methods:**

A retrospective, observational, single-center study, between January 2013 and December 2016, was conducted. Two renal end points (> 25% decrement in eGFR and > 20 mL/min/1.73 m^2^ decrement relative to the baseline) were assessed. To estimate the effect of UL-FABP on time to the first event, log-rank test was performed.

**Results:**

A total of 24 Japanese outpatients with human immunodeficiency virus receiving TDF were enrolled. The outcome each occurred in two patients during the follow-up period. UL-FABP levels ≥4.0 μg/g creatinine was significantly associated with > 25% decrement and > 20 mL/min/1.73 m^2^ decrement (*p* = 0.006 and 0.001, respectively).

**Conclusion:**

Based on our preliminary analysis, UL-FABP levels ≥4.0 μg/g creatinine predict renal dysfunction in patients receiving TDF with low Uβ2MG levels.

## Background

Renal dysfunction is recognized with increasing frequency among the non-infectious comorbidities associated with human immunodeficiency virus (HIV) infection. It is caused by a number of factors, and nephrotoxicity resulting from antiretroviral therapy (ART) is one of them. Tenofovir disoproxil fumarate (TDF) is known to reduce the estimated glomerular filtration rate (eGFR). Although the mechanism of tenofovir-induced kidney damage is not completely understood, mitochondrial toxicity in proximal renal tubular cells is considered the main cause [1]. In tenofovir-induced nephrotoxicity, tubular dysfunction is considered to precede the decline in eGFR, suggesting that tubular markers are more sensitive than eGFR in screening for nephrotoxicity in patients receiving TDF [2]. Liver-type fatty acid–binding protein (L-FABP) is also a tubular marker, and expressed in the proximal tubules of the human kidney and participates in fatty acid metabolism [3]. Urinary L-FABP (UL-FABP) level (≥ 4.0 μg/g creatinine) was a potential predictor of renal dysfunction in patients receiving ART in our previous pilot study [4]. However, it was not shown whether UL-FABP level was an independent risk factor for renal dysfunction or not because the pilot study was with too small a sample size to perform a multivariate analysis. In other words, it has not been known that UL-FABP was a risk factor for renal dysfunction regardless of whether Urinary β2 microglobulin (Uβ2MG) level was high or low. Uβ2MG is a well-known biomarker of TDF-related tubulopathy, and it was demonstrated that Uβ2MG levels ≥1700 μg/L were related to renal dysfunction in patients receiving TDF [5]. However, renal dysfunction has also occurred in patients receiving TDF with low Uβ2MG levels [5]. Thus, we focused on UL-FABP in patients receiving TDF with low Uβ2MG levels. The aim of this study was to gain a better understanding of the clinical utility of UL-FABP in patients receiving TDF with low Uβ2MG levels.

## Methods

### Study design and patient population

This study was a retrospective, single-centre cohort design using the medical chart review at The Hospital of Hyogo College of Medicine in Hyogo, Japan. The inclusion criteria were: patients with HIV who were ≥ 20 years old and received TDF at baseline; the UL-FABP level was measured between January 2013 and June 2014; the baseline eGFR was ≥ 60 mL/min/1.73 m^2^; the baseline eGFR was < 90 mL/min/1.73 m^2^. The following exclusion criteria were applied: patients who were not Japanese; the baseline Uβ2MG was ≥1700 μg/L [5]. Baseline was defined as the nearest date of the measurement of eGFR to the first measurement of UL-FABP level between January 2013 and June 2014.

### Follow-up evaluation

Patients were followed until December 2016. The end points were the following: more than 25% decrement in eGFR relative to the baseline [5]; and decrement in eGFR of more than 20 mL/min/1.73 m^2^ relative to the baseline [5]. Censoring occurred at the date of the discontinuation of TDF. Censoring was also performed on the day when ART including dolutegravir or cobisistat was switched to ART not including dolutegravir or cobisistat. Subsequently, censoring was performed on the day that ART not including dolutegravir or cobisistat was switched to ART including dolutegravir or cobisistat. Because dolutegravir and cobisistat have also been observed to apparently decrease eGFR based on serum creatinine of more than 10 mL/min/1.73 m^2^ without affecting the actual glomerular filtration rate [6–8], end points are greatly affected by the switch to or from dolutegravir or cobisistat. Finally, censoring was done at the end of the study period. The time of outcome was defined as the first date on which either the renal end points were observed.

### Anthropometric and laboratory evaluation

Non-fasting blood and spot urine samples were collected for analysis as part of routine clinical visits. The UL-FABP levels were measured by enzyme-linked immunosorbent assay (Renapro L-FABP test; CMIC Co., Tokyo, Japan; lower detection limit, 2.9 ng/mL), and were expressed as a ratio to urinary creatinine. UL-FABP levels below the lower detection limit were approximated using the lower detection limit. Uβ2MG was measured with a latex aggregation assay (BMG-Latex X1”Seiken”; DENKA SEIKEN, Tokyo, Japan). Serum creatinine levels were measured via an enzymatic method, and eGFR was calculated as eGFR (mL/min/1.73 m^2^) = 194 × serum creatinine (mg/dL)^-1.094^ × age^-0.287^ [9].

### Statistical methods

Patients were divided into two groups according to UL-FABP levels of 4.0 μg/g creatinine based on the previous study [4]. Kaplan-Meier analysis and a log-rank test were then performed to estimate the effect of UL-FABP on time to the end points. A probability value < 0.05 was considered significant. All analyses were conducted using SPSS statistics version 24.0 software (IBM, Tokyo, Japan).

## Results

### Patient characteristics

A total of 29 patients met the inclusion criteria. Of these, 5 patients were excluded by the exclusion criteria, and 24 patients were enrolled in the study. Table 1 summarizes the demographic and clinical characteristics of individuals enrolled in this study at baseline. There were no significant differences in the clinical characteristics between individuals with ≥ and < UL-FABP levels of 4.0 μg/g creatinine.

**Table 1:** Patient characteristics

	All Patients	Urinary L-FABP levels		*P* value
		≥ 4 μg/g creatinine	< 4 μg/g creatinine	
Patients, *n*	24	5	19	
Follow-up^a^, days	529 (351, 920)	294 (280, 383)	770 (403, 952)	0.017
Follow-up^b^, days	559 (351, 920)	294 (280, 490)	770 (403, 952)	0.030
Men, *n* (%)	24 (100)	5 (100)	19 (100)	–
Age, years	42 (37, 49)	54 (39, 58)	42 (36, 46)	0.101
Duration of receiving TDF, weeks	102 (38, 248)	88 (49, 317)	115 (38, 229)	0.859
Key drug				0.491
INSTI, *n* (%)	12 (50)	2 (40)	10 (52)	
PI, *n* (%)	7 (29)	1 (20)	6 (32)	
NNRTI, *n* (%)	5 (21)	2 (40)	3 (16)	
CD4 cell counts, cells/μL	571 (380, 790)	668 (529, 760)	557 (380, 818)	0.804
HIV-RNA level				1.000
< 20 copies/mL, *n* (%)	18 (75)	4 (80)	14 (74)	
20–500 copies/mL, *n* (%)	6 (25)	1 (20)	5 (26)	
Prior AIDS-defining illness, *n* (%)	4 (17)	0 (0)	4 (21)	0.544
eGFR, mL/min/1.73 m^2^	82.5 (78.7, 85.4)	80.5 (73.2, 85.4)	82.5 (79.1, 85)	0.414
Urinary β2MG level, μg/L	234 (122, 374)	344 (308, 957)	203 (122, 340)	0.214
Urinary L-FABP level, μg/g creatinine	1.8 (1.0, 4.0)	5.0 (4.4, 19.1)	1.4 (0.9, 2.8)	0.001
Haemophilia (+), *n* (%)	0 (0)	0 (0)	0 (0)	–
Diabetes mellitus (+), *n* (%)	0 (0)	0 (0)	0 (0)	–
Dyslipidaemia (+), *n* (%)	2 (8)	0 (0)	2 (11)	1.000
Hypertension (+), *n* (%)	2 (8)	1 (20)	1 (5)	0.380
HBV (+), *n* (%)	2 (8)	0 (0)	2 (11)	1.000
HCV (+), *n* (%)	0 (0)	0 (0)	0 (0)	–

Data are expressed as number (percentage) or median (25, 75% interquartile range).

^a^end point was more than 25% decrement in eGFR relative to the baseline.

^b^end points was decrement in eGFR of more than 20 mL/min/1.73 m^2^ relative to the baseline.

TDF tenofovir disoproxil fumarate, *INSTI* integrase strand transfer inhibitor, *PI* protease inhibitor, *NNRTI* non-nucleoside reverse transcriptase inhibitor, *AIDS* acquired immune deficiency syndrome, *eGFR* estimated glomerular filtration rate, *β2MG* beta-2 Microglobulin, *L-FABP* liver-type fatty acid-binding protein, *HBV* hepatitis B virus, HCV hepatitis C virus

### Survival curve of endpoint

**Fig. 1:**
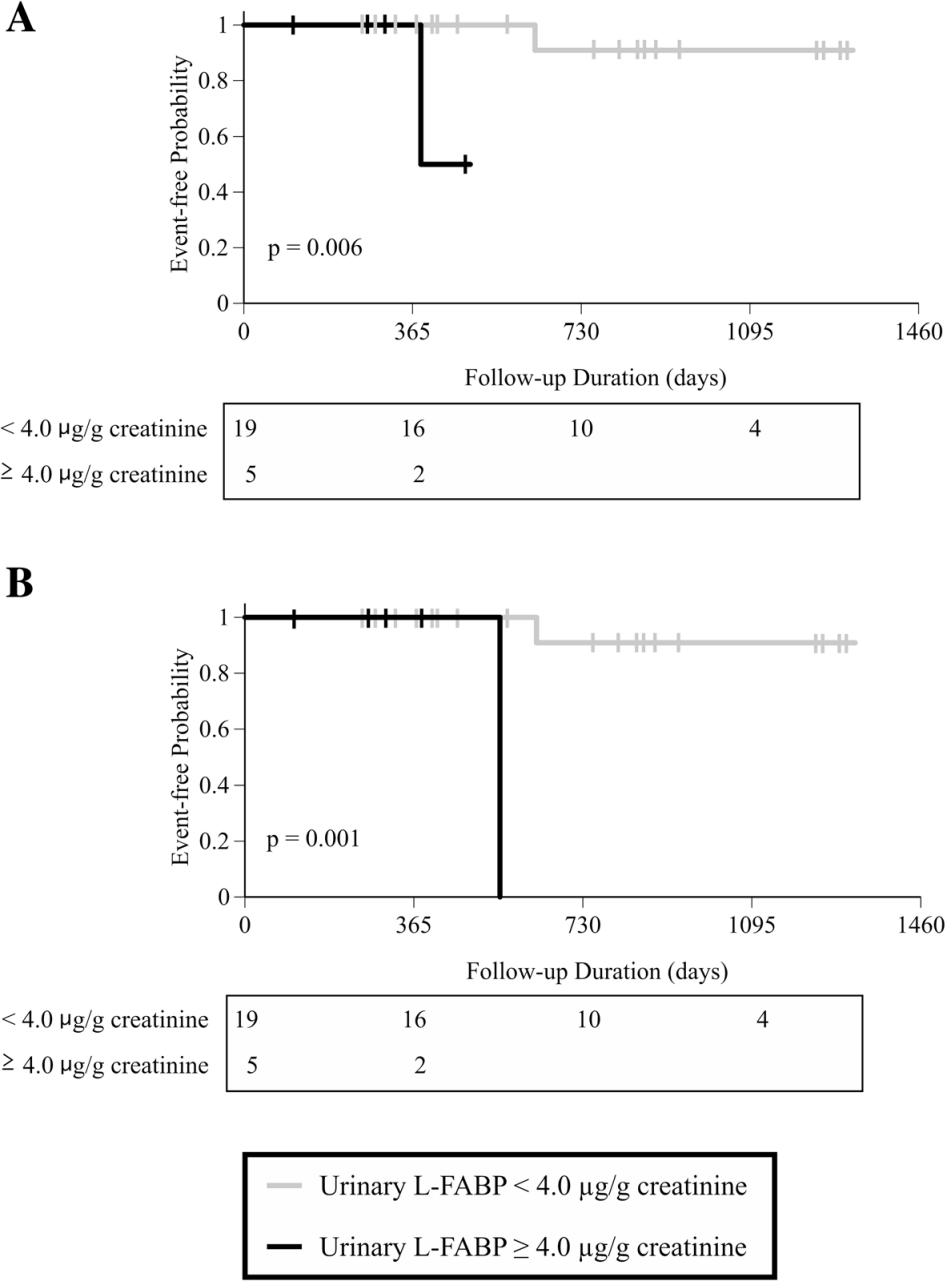
Kaplan–Meier curves for event-free survival of urinary L-FABP. Event was defined as (**a**) > 25% decrement and (**b**) > 20 mL/min/1.73 m^2^ decrement in eGFR. Patients were divided into two groups based on urinary L-FABP levels. The lower group is represented using the grey line and the higher group is represented by the black line. Differences between the higher and lower group are compared using a log-rank test *eGFR* estimated glomerular filtration rate, *L-FABP* liver-type fatty acid-binding protein.

The outcome each occurred in two patients during the follow-up period. Figure 1 shows the Kaplan-Meier survival probabilities for two end points based on the UL-FABP. The cumulative risk of more than 25% decrement in eGFR and decrement in eGFR of more than 20 mL/min/1.73 m^2^ relative to the baseline was higher in patients with higher UL-FABP levels (*p* = 0.006 and *p* = 0.001, respectively). The two patients who experienced more than 25% decrement in eGFR and decrement in eGFR of more than 20 mL/min/1.73 m^2^ were same. They did not receive an oral nephrotoxic drug expect for TDF.

## Discussion

Although UL-FABP level was suggested to be a potential predictor of renal dysfunction [4], availability of UL-FABP level in patients with low Uβ2MG level remains unclear. To our knowledge, this is the first study that showed the usefulness of UL-FABP in predicting eGFR decrement in patients receiving TDF with low Uβ2MG levels. Tubulointerstitial damage is considered to be the main cause of TDF-related renal dysfunction [1]. Among the tubular markers, Uβ2MG is a well-known biomarker of TDF-related tubulopathy [10]. Uβ2MG levels increase after the occurrence of proximal tubular structural injury. On the other hand, L-FABP is expressed in the proximal tubules, and it is an effective endogenous antioxidant during oxidative stress generated in pathophysiologic conditions [11]. Therefore, in early-stage renal dysfunction, it is possible that chronic ischemia and oxidative stress could have induced an increase in urinary excretion of L-FABP, even with low Uβ2MG levels.

UL-FABP levels ≥4.0 μg/g creatinine were risk factor for eGFR decrement in this study. UL-FABP levels above the normal upper limit (8.4 μg/g creatinine) were a risk factor for progression of diabetic nephropathy [12] and progression to ESRD [13]. However, a urinary L-FABP level below the normal upper limit had been observed in patients with microalbuminuria [14, 15]. Microalbuminuria is a widely recognized early marker of renal dysfunction [16]. These reports support our results that UL-FABP levels below the normal upper limit was a risk factor for renal dysfunction.

Our study has several limitations. First, the findings should be considered preliminary because this was a pilot study with too small a sample size to perform a multivariate analysis. Additional studies are needed to confirm these findings using a larger sample size. A longer follow-up period will give more predictable results and would confirm its validity. Second, eGFR was assessed using the formula of the Japanese Society of Nephrology [9] because the Chronic Kidney Disease Epidemiology Collaboration equation is limited by the differences in creatinine generation among ethnicities [9]. Therefore, these results may not be generalizable to non-Japanese populations.

## Conclusion

The present study demonstrates that UL-FABP levels predict renal dysfunction in patients receiving TDF with low Uβ2MG levels. Measurement of UL-FABP may be useful for the detection of patients at greater risk of progression to renal dysfunction due to TDF.

## Abbreviations

ART: Antiretroviral therapy
eGFR: Estimated glomerular filtration rate
HIV: Human immunodeficiency virus
L-FABP: Liver-type fatty acid–binding protein
TDF: Tenofovir disoproxil fumarate
UL-FABP: Urinary Liver-type fatty acid–binding protein
Uβ2MG: Urinary β2 microglobulin
